# RSM-Based Optimization Analysis for Cold Plasma and Ultrasound-Assisted Drying of Caraway Seed

**DOI:** 10.3390/foods13193084

**Published:** 2024-09-27

**Authors:** Moslem Namjoo, Nesa Dibagar, Hossein Golbakhshi, Adam Figiel, Klaudia Masztalerz

**Affiliations:** 1 Department of Mechanical Engineering of Biosystems, Faculty of Agriculture, University of Jiroft, Jiroft 7867155311, Iran; m.namjoo@ujiroft.ac.ir; 2 Institute of Agricultural Engineering, Faculty of Life Sciences and Technology, Wrocław University of Environmental and Life Sciences, 51-630 Wrocław, Poland; nesa.dibagar@upwr.edu.pl (N.D.); klaudia.masztalerz@upwr.edu.pl (K.M.); 3 Department of Mechanical Engineering, University of Jiroft, Jiroft 7867155311, Iran

**Keywords:** caraway seed, cold plasma, ultrasound, RSM, optimization

## Abstract

In this study, the hot-air drying of caraway seeds was enhanced using two nonthermal physical field technologies: cold plasma (CP) and ultrasonic waves (US). Air drying temperatures of 35, 45, and 55 °C with CP pretreatment exposure times (CP_t_) of 25 and 50 s were used. When convective drying was accompanied by US, power levels (US_p_) of 60, 120, and 180 W were applied. Experimentally, the most effective contribution was found by using both CP pretreatment (25 s) and US (180 W), in which the maximum decreases of 31% and 39% were estimated for the drying period and specific energy consumption, respectively. The total color change, the rupture force, *TPC*, *TFC*, and antioxidant capacity were also estimated for evaluating the quality of dried products. In a CP-US-assisted drying program (25 s, 180 W), the minimum change in color and the rupture force were found to be 6.40 N and 20.21 N, respectively. Compared to the pure air drying, the combined application of CP and US resulted in a mean increase of 53.2, 43.6, and 24.01% in *TPC*, *TFC*, and antioxidant capacity of extracts at the temperature of 35 °C. Based on the response surface methodology (RSM) approach and obtained experimental data, accurate mathematical predictive models were developed for finding the optimal drying condition. The optimization process revealed that 39 °C, 180 W, and 23 s resulted in a desirability of 0.78 for drying caraway seeds.

## 1. Introduction

Caraway (*Carum carvi* L.) is a small annual herbaceous plant from the family of Apiaceae and is a member of the parsley category. Crushed caraway seed has a strong, pleasant smell and causes a fairly bitter taste in the mouth. This spice has widespread flavoring applications in the food and bread industries [1]. The caraway seed is also praised for its outstanding therapeutic benefits. In traditional medicine, caraway has long been used for the treatment of toothache, jaundice, hypertension, and respiratory disorders [2]. Further health benefits of caraway have also been revealed in recent studies [3,4].

The flavor of fresh aromatic herbs usually diminishes quickly after harvesting, and they might not be stored for a long time [5,6]. Furthermore, the antioxidants and antimicrobial compounds in dried or ground spices are more abundant than those in the fresh material [7]. The common dehydration techniques for producing dry seeds are used in solar systems [8], oven dryers [9], freeze-drying [10], infrared systems [11], and microwave dehydrators [12]. However, long drying times and high amounts of incorporated energies in convective-type dryers may impose some detrimental effects on the appearance and structure of the products [13,14]. Therefore, conventional drying methods should be assisted by various new technologies to improve the dehydration process [15].

Plasma is the fourth state of matter, distinct from solid, liquid, and gas. In scientific terms, plasma is a highly ionized gas composed of charged particles, which can be a combination of ions (positively or negatively charged atoms) or electrons [16]. These charged particles exhibit collective behavior due to their interactions, resulting in unique properties. Cold plasma refers to a type of plasma that operates at lower temperatures compared to traditional high-temperature plasma. Unlike the extremely hot plasmas used in fusion reactors or cutting torches, cold plasma remains less well ionized. In cold plasma, the electrons may be at high temperatures, but the positive ions and neutral particles remain at lower temperatures [17].

Cold plasma (CP) technology has found various applications in the field of food science, offering innovative solutions for food safety and quality. Food microbial decontamination, functionalization, packaging, and waste treatment are among the applications of cold plasma [18,19]. Previous investigations demonstrated the contributing effects of cold plasma for destroying the spoilage bacteria, fungus species, and toxic compounds from the skin layer of fresh fruits and vegetables [20,21,22]. Cold plasma has also emerged as an innovative pretreatment technology in the field of food drying, offering several advantages. It improves the moisture diffusivity at the surface of the products and significantly decreases the drying time and the required intensity of the drying power [23]. As a result, fewer thermal effects are inflicted, and the physical as well as nutritional properties of food materials are preserved [24,25,26]. CP technology helps with lower energy consumption during drying. Overall, by optimizing moisture removal, CP contributes to more sustainable and cost-effective food drying [25]. Moreover, CP-dried foods are of higher quality. It achieves this by reducing enzyme activity, preserving nutritional content, and maintaining desirable sensory attributes [27]. Recently, many published studies have evidenced the positive contribution of cold plasma to the drying of seeds and herbs [16,23,24,25,26,27,28,29,30,31]. Ultrasound waves, in addition to cold plasma, play a crucial role in food dehydration. Unlike thermal drying techniques, ultrasound relies on sound waves with frequencies beyond human hearing. Specifically, for food drying, ultrasound waves operate at 20–26 kHz with an intensity exceeding 1 W/cm^2^. When applied during the drying process, these waves cause structural compression and expansion within the food, effectively squeezing out moisture and promoting drying [32]. The key advantage of ultrasound-assisted drying lies in its ability to minimize the reliance on excessive heat. As a result, it leads to faster drying rates, reduced energy consumption, improved food quality, and enhanced retention of bioactive compounds [15].

Conventional drying methods can negatively impact the sensory and nutritional quality of aromatic seeds. High temperatures during drying can lead to the loss of essential oils, resulting in reduced flavor and aroma. Additionally, heat-sensitive nutrients, such as vitamins and phenolic compounds, can degrade, lowering the nutritional value. The process can also cause undesirable color changes and a harder texture [7]. To alleviate these drawbacks, incorporating cold plasma and ultrasound assistance during conventional drying can be effective due to less thermal damage [33,34]. Cold plasma pretreatment prior to ultrasound-assisted air drying of cumin seeds significantly increased effective moisture diffusivity and reduced the drying time, energy consumption, and color change. In a study, an innovative strategy with cold plasma pretreatment followed by ultrasound-assisted convective drying was proposed for the drying of golden berries. This drying approach could significantly contribute to food security by improving product quality, nutritional value, and shelf stability, and reducing greenhouse gas emissions. In such a hybrid process, several more parameters are involved compared with a pure conventional drying method [33]. Therefore, to find the optimal processing conditions, numerous experiments are needed and huge amounts of obtained data should be interpreted. This is a very laborious and time-consuming study. In such a multivariate problem, some effective data-processing methods can provide reliable and accurate insight just from a limited number of experiments. Response surface methodology (RSM) is a statistical analysis method used for finding the optimum state when a process is affected by several variables [35]. In this approach, the results of well-designed experiments are used to reveal the optimal relationships between the factors [36].

Caraway’s historical significance and widespread use make it an interesting herb to explore in Kerman province, Iran. The popularity is based on local culinary traditions and individual preferences. With this overview, this study was undertaken to investigate the effect of single and parallel application of nonthermal and physical field technologies of cold plasma and ultrasound on the drying time, effective moisture diffusivity, specific energy consumption, color change, and rupture force. The effects of the included auxiliary sources (cold plasma and ultrasound waves) on the total phenolic content, total flavonoid content, and antioxidant capacity of caraway seeds are also studied. The study then determined optimal process conditions using the RSM.

## 2. Materials and Methods

### 2.1. Material

To gather appropriate specimens for the experimental research, a nearby farm situated 50 km from the laboratory in Kerman province, Iran, was chosen to collect fresh caraway seeds. The seeds were packed firmly to keep the initial moisture content. This also allowed for uniform distribution of moisture among all packed samples. Before any drying operation, the prepared samples were held at a temperature of 4 ± 1 °C for the maximum preservation of the initial quality of samples.

### 2.2. CP Pretreatment

**Figure 1:**
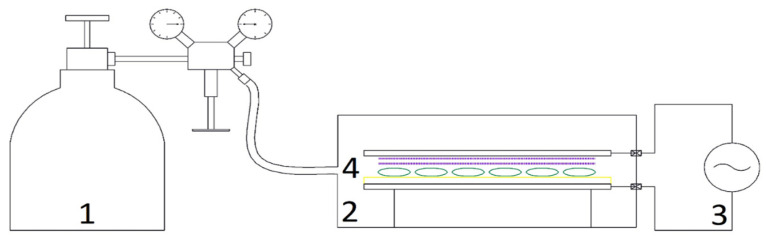
Schematic diagram of the CP experimental system: (1) gas supply control unit, (2) DBD reactor, (3) CP generator, and (4) sample seeds.

The CP device in the excitation mode of dielectric barrier discharge (DBD) was used to pretreat the seeds. Figure 1 shows the schematic view of the CP device and its main components. The distance between the two electrodes was 3.5 mm. A 6.2 kV input voltage, a 10 kHz pulse frequency, and a 300 W power were used to create the nitrogen gas (N_2_) plasma. A pretreatment of CP was investigated for approximately 60 g of caraway seeds at exposure durations of 25 and 50 s before each drying run. Following CP pretreatment, 10 g of the samples were separated to measure the moisture content and assess the color qualities of the pretreated seeds to make sure that the initial moisture content and color quality of the samples were not significantly changed.

### 2.3. Main Drying Procedure

**Figure 2:**
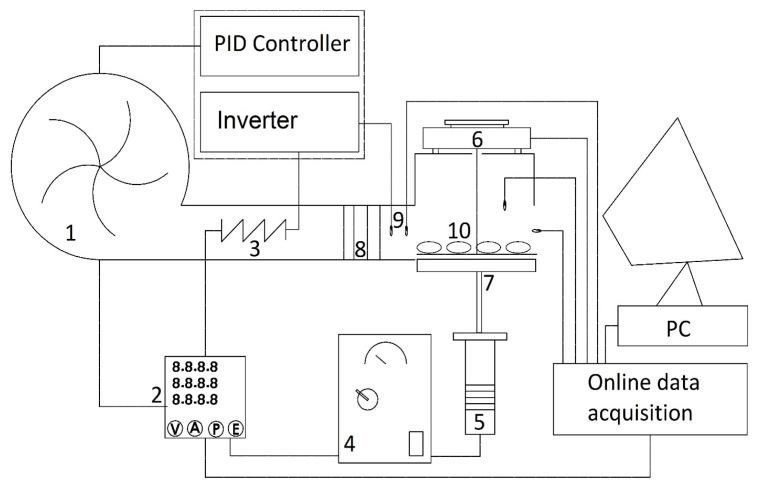
A schematic view of the combined hot-air/power ultrasound drying system: 1—centrifugal fan, 2—power meter, 3—thermal element, 4—ultrasonic generator, 5—ultrasonic transducer, 6—digital balance, 7—vibrating element (ultrasonic horn), 8—perforated chamber, 9—temperature and relative humidity sensor, and 10—drying samples.

All experimental tests were performed in a hybrid convective/ultrasound drying system, which is schematically depicted in Figure 2. This drying system was designed and fabricated at the Faculty of Agriculture, University of Jiroft, Jiroft, Iran. Its main parts were elaborately described by Namjoo et al. [5]. In this drying system, electrical heaters with the power of 3 kW were used to warm up the air. During the experiments, the heating element with the selected capacity could provide stable drying temperatures of 35, 45, and 55 °C inside the drying chamber. A centrifugal fan (BEF-14-7V2SP), which provided a discharge of 550 m^3^/h at 2200 rpm, circulated the heated air through the ducts and the drying chamber. An ultrasound unit (Farasot Zagros Co., Shahrekord, Iran), with a power of 1200 W and a frequency of 20 kHz, was selected as an auxiliary drying unit. A multivariable monitoring system always controlled the humidity, temperature, and sample weight during the experiments. This made it possible to automatically determine whether the samples attained the desired level of moisture content or whether the drying process should be continued. To measure and log the temperature and humidity data of the drying system, an Arduino temperature logger was developed.

### 2.4. Drying Kinetics and Physical Characterization

#### 2.4.1. Drying Time (*DT*) Determination

The drying experiments were conducted to obtain data for the moisture content of caraway seeds as a function of time. Samples with an initial moisture content of 56.3 ± 1% (d.b.) were dried to reach the final moisture level of about 10 ± 0.5% (d.b.). The required time was recorded as the drying duration in the selected drying program.

#### 2.4.2. Determination of Effective Moisture Diffusivity (*D_eff_*) 

The drying period for food-based materials is primarily influenced by the moisture transfer rate from the bulk to the skin surface. Therefore, in the second law of Fick, a term referred to as the effective moisture diffusivity (*D_eff_*) was introduced to evaluate the drying of various food textures.

By neglecting the transfer resistance at the skin surface and assuming a uniform decrease in moisture content, the following one-dimensional equation was obtained based on Fick’s considerations [37]:

(1) $$\frac{𝜕MR}{𝜕t}=\frac{𝜕}{𝜕x}\left(D_{eff}\frac{𝜕MR}{𝜕x}\right)$$

where *M* is the local moisture content (% d.b.), and *t* denotes the drying period. In the case of constant *D_eff_*, and considering a layer of caraway seeds with the geometry of a circular slab with a thickness of 5 mm, Park [37] found the following series solution for Equation (2):

(2) $$MR=\frac{8}{\pi^{2}}\sum_{n=0}^{\infty }\frac{1}{{\left(2n+1\right)}^{2}}\exp\left(-\frac{\pi^{2}}{4L^{2}}\left(2n+1\right)\cdot D_{eff}\cdot t\right)$$

where *MR* is the moisture ratio (g/g), *L* is the half-thickness of samples, and *n* is the number of drying terms that are taken into account for the solution. Equation (2) can also be treated as a diffusion drying model, which describes the decrease in *MR* with respect to the drying time [9]. The moisture ratio (*MR*) of caraway seeds was obtained by Equation (3) during drying:

(3) $$MR=\frac{M_{t}-M_{e}}{M_{0}-M_{e}}$$

where *M* is the moisture content (% d.b.), and subscripts *t*, *e*, and 0 describe the instantaneous, equilibrium, and initial values, respectively.

The food materials usually have a prolonged drying time, and the higher-order terms in (2) may be neglected [37]. As a result, the solution simply reduces to:

(4) $$\ln\left(MR\right)=\ln\left(\frac{8}{\pi^{2}}\right)-\left(\frac{\pi^{2}}{4L^{2}}D_{eff}\right)\cdot t$$

As can be seen, a linear regression (y = m*x* + b) can be found for the natural logarithm of *MR*, and the slope, *m*, found from plotting the experimental data yielded the following equation for the effective diffusivity:

(5) $$D_{eff}=\frac{m\times 4L^{2}}{\pi^{2}}$$

#### 2.4.3. Determination of Specific Energy Consumption (*SEC*) 

To achieve the desired level of moisture content, some amount of electrical energy should be supplied for blowing fans, heaters, and ultrasound generators. The sensors and controlling unit also need a minor amount of energy to operate.

**Figure 3:**
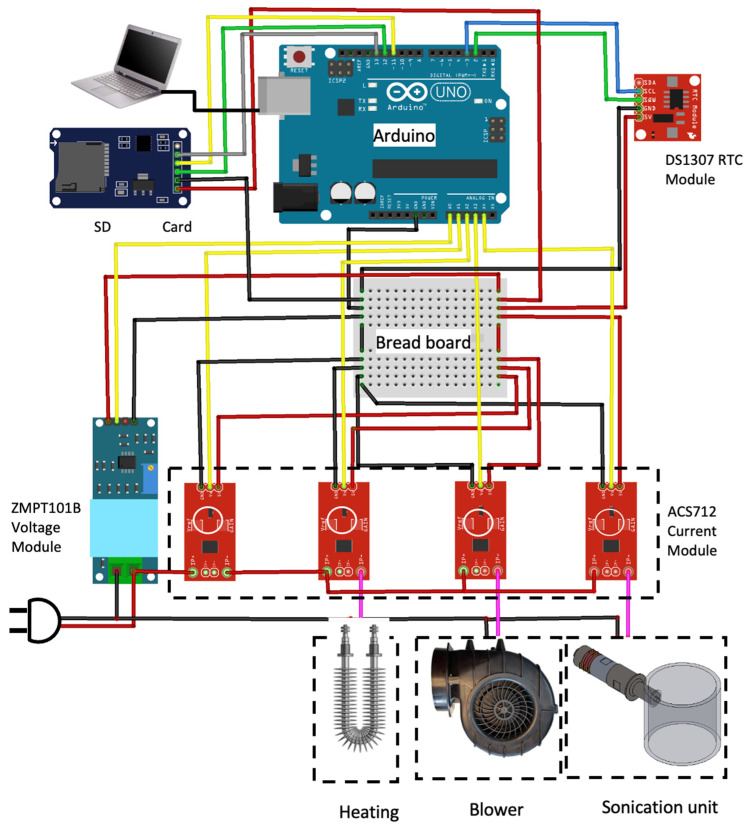
Actual wiring between the Arduino UNO board, RTC, voltmeter, ammeter, and micro-SD card modules designed to measure the energy consumption of the drying system.

To measure and manage the energy consumption of the drying system, a new kind of energy monitoring system was utilized consisting of an Arduino UNO, Real-Time Clock (RTC), voltmeter, ammeter, and micro-SD card modules. The main board was Arduino UNO, which is an 8-bit microcontroller based on the ATmega328 (Microchip Technology Inc., Chandler, AZ, USA). The instantaneous and precise measurement of the applied voltage and electric current in the drying system was carried out by ACS712 and ZMPT101B modules (Xin Hui Electronic Technology Co., Ltd., Guangzhou, Guangdong, China), respectively. The ZMPT101B module measures the AC main voltage up to 250 volts. Therefore, it can be widely used as a sensing element in industrial equipment to measure the supply of voltage. The ACS712 current module consists of a precise, low-offset, linear Hall sensor circuit with a copper conduction path, located near the surface of the die. Applied current flow through this copper conduction path generates a magnetic field, which is sensed by the integrated Hall IC and converted into a proportional voltage. According to Figure 3, four ACS712 current modules were provided to measure the current flow of the ultrasonic unit, blower, heating unit, and the entire drying system, separately. The DS1307 (Xin Hui Electronic Technology Co., Ltd., Guangzhou, Guangdong, China) is an RTC module that was used to maintain the date and time for most of the electronic projects. This module is interfaced with Arduino using the I2C communication (SCL and SDA), and the SD card module is interfaced using the SPI communication (MISO, MOSI, SCK, and CS). Since the current flow time from each electric current module was measured by the DS1307 module, three factors, including time, instantaneous voltage, and current, were provided to the Arduino microcontroller. For calculating the real-time energy consumption of the drying system, individual codes were written and complied with the Arduino IDE. Further, a Delta power meter (Ziegler Co., Schwäbisch Hall, Baden-Württemberg, Germany) was simultaneously employed to measure the energy consumption and evaluate the accuracy of the data obtained from the utilized energy metering system (Figure 3).

Specific energy consumption (MJ/kg) of a dehydration process is an alternative estimate of the dryer efficiency and was calculated using Equation (6):

(6) $$SEC=\frac{E_{t}}{m_{w}}$$

where *E_t_* is the total energy consumed during the drying process (MJ), and *m_w_* is the mass of the vaporized water from the product during drying (kg).

#### 2.4.4. Determination of Color Change (*ΔE*)

A quantitative determination of the color quality of samples provides a suitable criterion for evaluating the effectiveness of selected conditions for the drying system. In this study, the total change in the color was estimated by comparing the images taken from fresh and dried seeds. The images were taken by a digital camera, which at each pixel had three detectors for red, green, and blue colors. A chamber fabricated from medium-density fiberboards (MDF) was used to take photographs of the seeds. The internal surfaces of the chamber were coated with clear, sleek glass. By this consideration, the light of an LED lamp placed inside the chamber was reflected uniformly from all surfaces, and the photographs were taken without any shadow.

Using the algorithms included in MATLAB R2017a, the images were processed, and three parameters were defined for numerical assessment of the color quality of samples. In this regard, L* was defined as describing the darkness or whiteness of the seed’s image. The greenness or redness of samples was evaluated by the amount of a* and, finally, the blueness or yellowness of seeds was estimated from the value attributed to b*. Then, the total change in the color quality (*ΔE*) was found from the following equation [38]:

(7) $$\Delta E=\sqrt{{\left(L^{*}-L_{0}^{*}\right)}^{2}+{\left(a^{*}-a_{0}^{*}\right)}^{2}+{\left(b^{*}-b_{0}^{*}\right)}^{2}}$$

where *L*_0_, *a*_0_, and *b*_0_ are the values estimated for the fresh seeds, and L*, a*, and b* are found from image processing of dried samples. So, the higher values of *ΔE* clearly demonstrated a significant degradation in the color quality after the drying process.

#### 2.4.5. Determintion of Rupture Force (*RF*)

The rupture force is the minimum amount of compressive load exerted for crushing the samples. This force is estimated by evaluating the area under the curve in the force–displacement diagram [39]. In this study, the mechanical tests were performed using a Universal Instron Machine (STM-20, SANTAM Co., Tehran, Iran). For measuring the applied force and endured deformation, a compressive load cell (DBBP-20, BONGSHIN Co., Incheon, Republic of Korea) with accuracies of ±0.01 N and ±0.001 mm for force and displacement, respectively, was installed in the testing machine. The seed was placed between the two horizontal plates of the apparatus, which had a relative approaching velocity of 2 mm/min. The variations in the resisting compressive force were recorded by a data logger until the rupture point. Then, at each treatment, the results were used to plot a load–displacement curve for determination of rupture force. For evaluating a more reliable estimation, 10 runs were considered for each drying condition.

### 2.5. Biochemical Characterization

#### 2.5.1. Extract Preparation

In this study, the method of Mau et al. (2001) was slightly modified for preparing the extracts. Briefly, dried caraway seeds were ground into powder using a miller (Ika-Werke, Staufen, Germany). Then, 2.5 g of powder was mixed with 25 mL of 60% methanol as the extraction solvent and well stirred for 30 min. The resulting extract was stored in a refrigerator at 4 °C and, after 24 h, was filtered through Whatman No. 4 filter paper. Then, the extracts were evaporated to dryness under vacuum. The obtained crude extract was stored at 4 °C for future use [40].

#### 2.5.2. Determination of Total Phenolic Content (*TPC*)

To evaluate the total phenolic content (*TPC*), 0.1 mL of obtained extract was mixed with 0.125 mL of Folin–Ciocalteu reagent and 0.5 mL of distilled water. Then, 1.25 mL of 7% sodium carbonate solution (Na_2_CO_3_) was added, and the resulting mixture was diluted with distilled water to obtain a solution with a 3 mL volume. The mixture was vortexed and then allowed to stand for 90 min. Finally, a UV/visible spectrophotometer was used to measure the amount of absorbance at a level of 760 nm. The results for TPC were reported in milligrams (mg) of Gallic acid equivalents (GAE) per gram (g) of dried sample [41].

#### 2.5.3. Determination of Total Flavonoid Content (*TFC*)

To evaluate the total amount of flavonoid content (*TFC*), 1 mL of the extract was combined with 5 mL of distilled water and 0.3 mL of a 5% NaNO_2_ solution. After 6 min, 0.6 mL of a 10% AlCl_3_·6H_2_O solution was added. After 5 min, the mixture was combined with 2 mL of NaOH (1 M) and then diluted with distilled water to a solution with a total volume of 10 mL. Immediately, a spectrophotometer was used to measure the absorbance at a level of 510 nm [42]. The results were expressed in milligrams of catechin equivalents (CE) per gram of dry weight (DW).

#### 2.5.4. DPPH Assay

A mixture of 0.25 mL of DPPH methanol solution and 1 mL of each sample was vigorously shaken for 1 min, and then left to stay in a dark room at 37 °C. Then, using a UV spectrophotometer, the absorption was measured at 517 nm to evaluate the antioxidant activity [43]. For indicating the concentration needed to achieve 50% inhibition, the results were expressed as *IC_50_* (micrograms per milliliter). A lower *IC_50_* value indicates higher antioxidant activity of the plant extract.

### 2.6. Response Surface Methodology (RSM)

RSM was applied for several purposes, including experimental design, data collection, model fitting, model validation, surface visualization, and optimization.

#### 2.6.1. Experimental Design and Data Collection

In this study, two sets of variables were introduced for caraway seeds’ drying. CP exposure time (*X*_1_), hot-air temperature (*X*_2_), and US power (*X*_3_) were the independent variables. The duration of the drying process, effective moisture diffusivity, amount of consumed energy, total color change of products, rupture force, TPC, TFC, and antioxidant activity were also defined as the response variables for evaluating the various designed drying variants. In the experimental design, the data matrix dimension depended on the specific experimental design used. Central composite design (CCD) was implemented since it was suspected that only significant factors remained after initial experimentation. The data matrix for CCD included:

Factorial points: These are the same as in the factorial design.

Center points: These are used to estimate curvature effects.

Axial (star) points: These are located at a distance from the center along each factor’s axis.

Three repetitions were considered for each factorial, axial, and center point.

#### 2.6.2. The Regression Analysis for the Experimental Data

In this study, the impact of input parameters was evaluated on the output variables. To analyze the regression and plot the response surfaces, RSM-based software “Design Expert 13.0.0” was used. In this way, sufficient data could be produced by the least number of runs. Through the following second-order polynomial, RSM provided accurate predictions for each dependent (response) variable, *Y*:

(8) $$\begin{array}{c} Y=B_{0}+B_{1}X_{1}+B_{2}X_{2}+B_{3}X_{3}+B_{11}X_{1}^{2}+B_{22}X_{2}^{2}+B_{33}X_{3}^{2}\\ +B_{12}X_{1}X_{2}+B_{13}X_{1}X_{3}+B_{23}X_{2}X_{3} \end{array}$$

Here, drying time, effective moisture diffusivity, total color change, rupture force, TPC, TFC, and antioxidant activity were the main responses. The drying temperature, ultrasound power, and the CP pretreatment time, as the input parameters, are designated in Equation (8) by *X*_1_, *X*_2_, and *X*_3_, respectively. The coefficients in Equation (8) are the main outputs of the RSM analysis, and the accuracy of regression depends on their value. As can be observed, *B_0_* is the constant term, *B*_1_, *B*_2_, and *B*_3_ show the linear effect of independent variables, and *B*_11_, *B*_22_, *B*_33_, *B*_12_, *B*_13_, and *B*_23_ yield the quadratic and interactive effects of *X*_1_, *X*_2_, and *X*_3_ on the response variables, respectively.

#### 2.6.3. Modeling and Optimization

In this study, empirical models were developed by RSM to describe the relationship between the independent variables and the responses. The following assumptions were considered in constructing a reliable model to explore the relationships between the research input variables and the responses, and to find the optimal conditions for the desired outcomes:

- The responses (dependent variables) are a function of the independent variables, often represented by a polynomial model, typically a second-order polynomial.
- The independent variables are continuous and can take on any value within a specified range. They are also assumed to be independent of each other, meaning the effect of one factor does not depend on the levels of other factors.
- The error term (residuals) is normally distributed with a mean of zero and constant variance.
- The model typically includes linear and quadratic terms to capture the curvature in the response surface, and interaction terms may also be included to account for the combined effect of two or more input variables.

The objective of optimization was to minimize the drying time, energy consumption, color change, and rupture force, while maximizing effective moisture diffusivity as well as the amounts of TPC, TFC, and antioxidant activity in the final products. Independent variables influencing the response were identified, and their ranges were determined based on the experimental results. The fitted model was used to generate response surfaces and contour plots, which were analyzed to identify the optimal conditions for the desired outcome.

#### 2.6.4. Validation

The validity of RSM models was investigated via comparison of experimental data with the results of polynomials in the form (8) proposed for each dependent variable. For 30% of performed tests, the results of the RSM model were also calculated for evaluating the accuracy of predictions. Then, based on the experimental value at the *i*th test, *Y^i^_exp_*, the RSM predicted value, *Y^i^_pre_*, and mean values ${\bar{Y}}_{\exp}$ and ${\bar{Y}}_{pre}$, the following statistical indicators were evaluated for each response variable, *Y* [38]:

(9) $$R^{2}=\frac{\sum_{i=1}^{n}\left(Y_{pre,i}-Y_{pre})(Y_{\exp,i}-Y_{\exp}\right)}{\sqrt{\sum_{i=1}^{n}{\left(Y_{pre,i}-Y_{pre}\right)}^{2}\sum_{i=1}^{n}{\left(Y_{\exp,i}-Y_{\exp}\right)}^{2}}}$$

(10) $$RMSE={\left[\frac{1}{n}\sum_{i=1}^{n}{\left(Y_{pre,i}-Y_{\exp,i}\right)}^{2}\right]}^{\frac{1}{2}}$$

(11) $$MAPE=\frac{1}{n}\sum_{i=1}^{n}\left|\frac{Y_{pre,i}-Y_{\exp,i}}{Y_{\exp,i}}\right|\times 100$$

(12) $$MAE=\frac{1}{n}\sum_{i=1}^{n}\left|Y_{pre,i}-Y_{\exp,i}\right|$$

where *R*^2^ is the correlation coefficient, and *RMSE* is the root mean square error (Equations (9) and (10)). The mean absolute percentage error is denoted by *MAPE*, and *MAE* stands for the mean absolute error (Equations (11) and (12), respectively). Higher values of *R* indicate a good correlation between the results of RSM models with the measured data, and lower values of *RMSE*, *MAPE*, and *MAE* demonstrate that the mathematical model can predict the test results with negligible errors.

### 2.7. Statistical Analysis

The statistical toolbox of RSM-based software “Design Expert 13.0.0” was employed for assessing the significance of the input variables and their interactions on the response variables. The analysis of variance (ANOVA) was used to decompose the total variation in the response into components attributable to different sources: the model, the residual error, and the lack of fit.

## 3. Results and Discussion

### 3.1. Statistical Analysis Results

Table 1 shows the full factorial design of experiments used in this research, along with the experimental response values. The results of the ANOVA, including the significant linear, quadratic, and interactive effects of the input variables (CP exposure time, convective air-drying temperature, and ultrasound power) on each response variable, are also depicted in Table 2. By excluding the non-significant coefficients in Equation (8), the final polynomial equations for each response variable are presented in Table 3.

### 3.2. Drying Time (DT)

**Figure 4:**
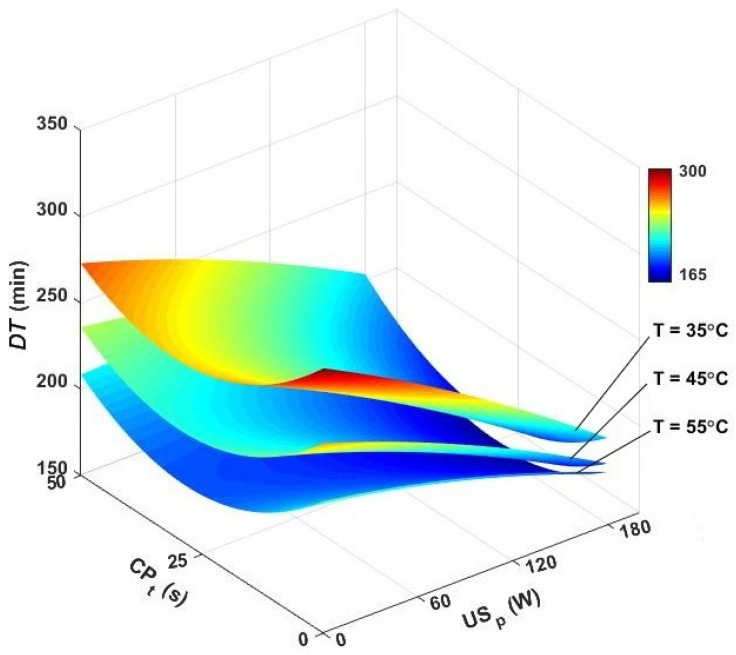
3D contour plots for drying time versus air temperature, cold plasma pretreatment, and ultrasonic power. *DT*: drying time, CP_t_: cold plasma exposure time, US_p_: ultrasound power.

The drying time of caraway seeds was assessed under different drying temperatures, ultrasound powers, and CP pretreatment times, with the results displayed in Figure 4. In the single air drying, the drying duration was in the range of 215–292 min depending on the temperature. The fastest drying (215 min) was achieved at 55 °C, while the longest drying duration (292 min) belonged to the temperature of 35 °C. Numerous studies have confirmed the significant impact of hot-air temperature on the drying duration of food materials. Higher temperatures generally accelerate the drying process by enhancing the rate of heat transfer and reducing the surface tension of water, which facilitates faster evaporation [44]. This relationship has been consistently observed across various types of food products, including cumin seeds during hot-air drying [34,45].

Following ultrasound assistance during hot-air drying, the drying time ranged from 181 to 273 min. In the US-assisted hot-air-drying program, the shortest drying time (181 min) was achieved at 45 °C with an ultrasound power of 180 W, while the longest drying time (273 min) occurred at 35 °C with an ultrasound power of 60 W. When the air drying is assisted by ultrasonic waves at the frequency of 20 kHz, the particles are forced to vibrate 20,000 times/s. This creates great intercellular and extracellular structural effects and significantly improves the internal and external transfer of moisture from the samples, resulting in a shorter drying time [15,46].

The findings also indicated that increasing the ultrasound power can further enhance water removal from the seeds, thereby reducing the drying time. At higher powers, the decay of ultrasonic wave amplitudes reduced, and more energy was conveyed to the sample [15]. The sonication did not increase the temperature of the drying material significantly. In many studies, the temperature rise caused by ultrasound waves did not exceed 1–2 °C [46,47].

Exposing caraway seeds to cold plasma for 25 s before convective drying reduced the drying time by 9.59, 9.84, and 8.37% at 35, 45, and 55 °C, respectively. Researchers used CP pretreatment before drying grapes, wolfberry, and saffron, and they observed the positive contribution of plasma jet to improving the drying rate and diminishing drying duration [24,29,31]. The CP pretreatment stimulated the electrons and ions and generated holes and cracks in the micron-scale on the skin layer of samples. This greatly improved the moisture removal and drying rate [29,30]. While CP pretreatment alone was less effective than ultrasound-assisted drying, combining both technologies produced even better results. The drying time for caraway seeds by CP and ultrasound assistance ranged from 135 to 248 min, significantly shorter than using hot-air drying alone. The shortest drying time (135 min) was achieved with a CP_t_: 25 s, followed by hybrid processing at 45 °C and 180 W. In contrast, the longest drying time occurred with a CP_t_: 50 s, 60 W ultrasonic power, and 35 °C convective temperature, suggesting that prolonged CP exposure can cause surface hardening and increase evaporative resistance. Studies have indicated that excessive CP exposure can disrupt cell walls, making water removal more difficult [48,49]. Figure 4 shows that a 25 s CP exposure is an effective pretreatment, resulting in an average 30% reduction in drying time when used with ultrasound assistance.

### 3.3. Effective Moisture Diffusivity (D_eff_)

**Figure 5:**
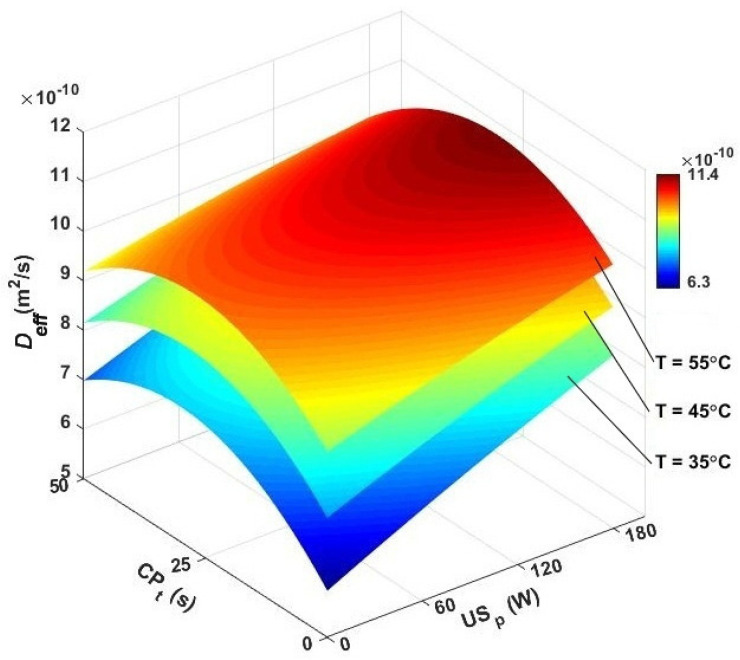
3D contour plots for effective moisture diffusivity against air temperature, cold plasma pretreatment, and sonication power. *D_eff_*: diffusivity, CP_t_: cold plasma exposure time, and US_p_: ultrasound power.

The impact of the independent variables on improving the effective moisture diffusivity of samples is illustrated in Figure 5. In hot-air drying, by increasing the temperature, the effective diffusivity of seeds increased, and the maximum value of 9.01 × 10^−10^ m^2^/s was found at 55 °C. The minimum diffusivity at 35 °C was noted to be 6.55 × 10^−10^ m^2^/s. In other related works, it has been confirmed that higher air temperatures can improve the moisture diffusion of dried samples [12,50,51].

In US-assisted hot-air drying by utilizing different ultrasound powers, the moisture diffusivity increased and was found within a range of 7.03 × 10^−10^–1.02 × 10^−9^ m^2^/s. The maximum *D_eff_* (1.02 × 10^−9^ m^2^/s) was obtained at 55 °C, and the power of the ultrasound wave at 180 W, while the lowest diffusivity (7.03 × 10^−10^ m^2^/s) corresponded to 60 W and 35 °C. The acoustic energy generated repeating compressions and decompressions, referred to as the sponge effect, and significantly intensified the movement of moisture to the skin. As a result, the removal of water content to the drying air became easier [52,53,54]. For a constant ultrasound power, different contributions may be achieved at various air temperatures. Figure 5 shows that by activating an ultrasound wave with 180 W, the increase in effective moisture diffusivity of samples at 35, 45, and 55 °C was 28.24, 21.37, and 13.08%, respectively. This clearly indicated that at higher temperatures, the contributing effect of sonication diminished. It was noted that at higher temperatures, the air density was reduced, and less ultrasound energy could be conveyed to the sample [52].

In the CP-assisted hot-air-drying program, exposing caraway seeds to CP_t_: 25 s improved the effective moisture diffusivity of the samples by 10.84, 11.48, and 9.76% at 35, 45, and 55 °C, respectively. Recently, the positive effect of CP pretreatment has been reported for the drying of grapes [24] and jujube [16]. However, it is noted from Figure 5 that the sonication during hot-air drying provided more contribution to increasing *D_eff_* at the same air temperatures. The exposure time of 50 s provided even less modification for the diffusivity. This implies that over-exposing the products to CP significantly increased the diffusion resistance at the surface of the seed [25,30]. Figure 5 illustrates how the drying air temperature, ultrasound power, and CP exposure time influenced diffusivity. In the CP-US-assisted drying domain, the results for the effective diffusivity were recorded between 7.75 × 10^−10^–1.21 × 10^−9^ m^2^/s. The highest diffusivity (1.21 × 10^−9^ m^2^/s) was observed with a 25 s pretreatment, a drying temperature of 55 °C, and an ultrasound power of 180 W. Conversely, the lowest diffusivity (7.75 × 10^−10^ m^2^/s) was recorded with a 50 s CP exposure, followed by a drying temperature of 35 °C and an ultrasound power of 60 W.

### 3.4. Specific Energy Consumption (SEC)

**Figure 6:**
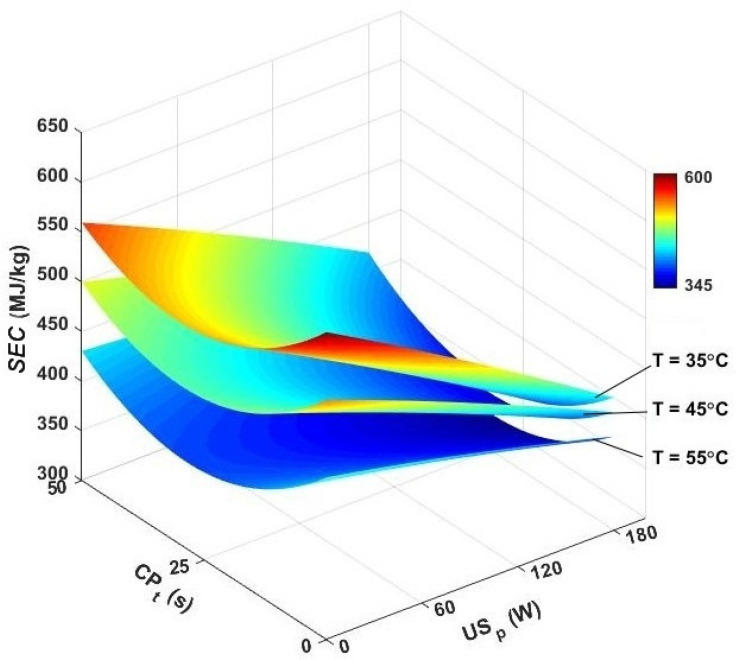
Effect of air temperature, cold plasma pretreatment, and sonication power on the specific energy consumption of caraway seeds during drying. *SEC*: specific energy consumption, CP_t_: cold plasma exposure time, and US_p_: ultrasound power.

The specific energy consumption is another useful criterion that can be used to evaluate the performance of a hybrid ultrasound/convective system in drying caraway seeds. Trying to minimize the specific energy consumption is a critical challenge in the design of any drying process. In the present research, the initial water content of samples was selected as 56.3% (d.b.), and they were held in the constructed drying setup until the desired level of 10% (d.b.). Under different drying conditions, the results for specific energy consumption are presented in Figure 6. In the convective drying system, the specific energy consumption varied between 457.79 and 582.47 MJ/kg. Wang et al. reported similar values for the drying of chrysanthemum by a hot-air dryer (331.20–486.36 MJ/kg) and by a microwave-assisted convective dryer (404.64–4642.56 MJ/kg) [55]. According to the results, the specific energy consumption at the air temperature of 55 °C was found to be 27.24% lower than that found at 35 °C. This is due to the fact that the warmer air has more absorbing capacity and can remove the desired amount of moisture in a shorter time. However, because of the adverse effects on the quality of products, the drying air temperature may not be un-limitedly increased. Therefore, it is preferable to minimize specific energy consumption by applying nonthermal treatments. In the ultrasound-assisted drying system, a range of about 401.1–551.58 MJ/kg was found for the specific energy consumption. The maximum specific energy consumption (551.58 MJ/kg) was reported at 35 °C and an ultrasound power of 60 W, whereas at 55 °C and 120 W, the minimum amount of *SEC* (401.1 MJ/kg) was utilized for drying seeds. In several other investigations, it was also reported that the ultrasound can increase the diffusion rate and lower the specific energy consumption [16,51,56].

In another drying process, the caraway seeds were first exposed to CP and then were dried in a hybrid ultrasound/convective system. In this program, the amount of specific energy consumption was considerably lower than what was observed in other drying variants, and the results were in a range of 303.47–517.43 MJ/kg. The maximum specific energy consumption (517.43 MJ/kg) was recorded when the seeds were pretreated with CP for 50 s, dried at 35 °C, and affected by an ultrasound wave with a power of 60 W. However, in drying with the 25 s CP pretreatment, drying air temperature of 55 °C, and 180 W sonication power, the minimum electrical power (303.47 MJ/kg) was used. The positive effects of CP pretreatment on reducing energy consumption were also reported for the drying of saffron [29] and grape [24]. According to Figure 6, after 25 s of pretreatment by CP, the evaluated amount of energy savings, compared to the pure hot-air-drying system, was 6.35, 9.82, and 10.65% at 35, 45, and 55 °C, respectively. It was noted that the contribution of pure cold plasma pretreatment was relatively lower than what was observed in the hybrid ultrasound/convective drying. Therefore, we realized that incorporating both CP pretreatment and the ultrasound effect provided a highly energy-efficient drying method for caraway seeds.

It is worth noting that all of the treatments and drying were performed in a thin layer and with the use of laboratory-scale equipment, which significantly affected the effectiveness of the process and consequently led to higher values of specific energy consumption in the study, compared with industrial-scale equipment. However, the results still showed important trends in specific energy consumption that can be extrapolated for larger-scale settings and provided important insight into the effect of ultrasound and cold plasma treatment on overall drying kinetics [57,58].

### 3.5. Total Color Change (ΔE)

**Figure 7:**
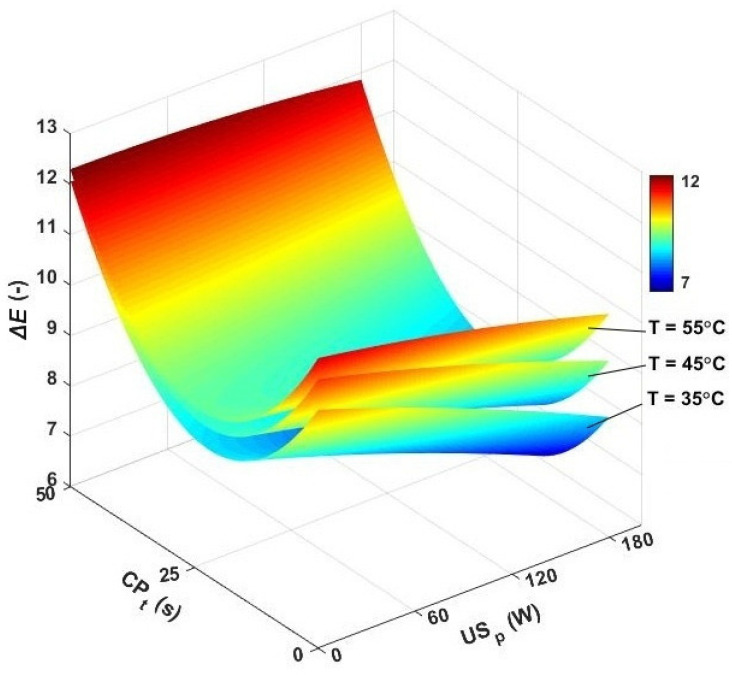
3D contour plots for total color change versus air temperature, cold plasma pretreatment, and ultrasonic power. *ΔE*: color change, CP_t_: cold plasma exposure time, and US_p_: ultrasound power.

In any drying procedure, a fraction of the compounds in the products are oxidized; especially, exposure to the hot circulating air may intensify the harmful effects on the natural color of seeds [24]. So, the degradation of the sample’s quality in a prolonged drying process seems to be inevitable [51]. In this section, the changes in the natural color of caraway seeds were investigated, and the results are presented in Figure 7. In the convective dryer, the color change of samples was only influenced by the temperature of the drying air. The minimum color change at 35 °C was evaluated as 10.79, while at 55 °C the total color change obtained its maximum value (11.42). By incorporating the ultrasound power, a lower range was found for the total color change of samples, and the results were found in a range of 8.47 to 11.15. The minimum color change (8.47) was observed for drying the seeds at 35 °C and utilizing an ultrasound power of 180 W, while the air temperature of 45 °C and the ultrasound power of 60 W induced the maximum color change (11.15) in the samples. The high-power sonication reduced the drying time and resultantly diminished the harmful effect of drying conditions on the natural color compounds of the samples. The contributions of ultrasound waves to the color quality have also been investigated for green pepper [59], paddy [46], and sunflower seeds [51].

The effect of cold plasma is also presented in Figure 7. For the seeds dried with a 25 s CP pretreatment, the total color change at 35, 45, and 55 °C was found to be 21.78, 19.88, and 16.81%, respectively. The observed contribution was higher than the color quality preservation achieved by applying ultrasound power. However, Figure 7 shows that 50 s of CP pretreatment had more adverse effects on the total color change of dried products. Zhang et al. argued that long CP pretreatment can damage the cell structure and degrade bioactive compounds in products [30]. Pankaj and Keener also reported that the increased CP time deteriorated the color quality of dried seeds [60].

The effect of the incorporation of both CP pretreatment and ultrasound was also investigated (Figure 7). As can be seen, for 25 s of CP pretreatment time and drying at different air temperatures and sonication powers, the color change of dried samples was reduced to only 6.4–12.1. Using both CP_t_: 25 s and ultrasound was noted to be the most sufficient program for preserving the quality of seeds. The lowest effect on total color change (6.4) occurred when drying with CP_t_: 25 s, US_p_: 180 W, and an air temperature of 35 °C. The maximum color change (12.1) in the pretreated caraway samples was observed after CP_t_: 50 s and drying at 55 °C and 60 W. So, by avoiding excess exposure to CP, and drying at lower temperatures with high sonication power, the highest color preservation may be achieved.

### 3.6. Rupture Force (RF)

**Figure 8:**
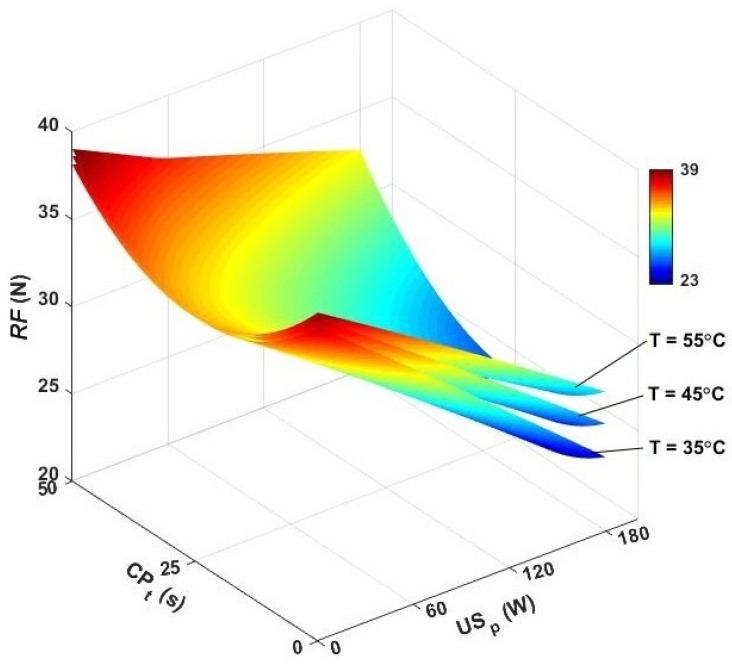
3D contour plots for rupture force versus air temperature, cold plasma pretreatment, and ultrasonic power. *RF*: rupture force, CP_t_: cold plasma exposure time, and US_p_: ultrasound power.

The rupture force is another index for evaluating the physical quality of foodstuffs. This parameter is defined as the compressive force required for crumbling the samples and is equivalent to the crispiness and brittleness of seeds after the drying process [61]. The effects of drying temperature, as well as exposing the seeds to ultrasound and CP pretreatment, were evaluated, and the results for the rupture force are presented in Figure 8. The rupture force in single hot-air drying was recorded within 38.02–38.16 N. The ANOVA results revealed that the temperature did not have a significant effect on improving the crispiness of dried samples.

The incorporation of ultrasound caused the collapse of cells and pores and resulted in the destruction of the food texture. In US-assisted hot-air drying, the results were found in a range of 25.38–35.37 N. The lowest value (25.38 N) was found at 35 °C and 180 W, and the maximum rupture force (35.37 N) was estimated at the temperature of 55 °C and ultrasound power of 60 W. According to the experimental observations, the ultrasound power of 180 W reduced the rupture force by 33.25, 28.41, and 23.56% at 35, 45, and 55 °C, respectively. According to the results, the high-power sonication resulted in the production of dried seeds with a significantly reduced rupture force. The contribution of ultrasound waves to the crispiness of the food texture has been previously elaborately investigated by researchers [62,63,64].

Cold plasma pretreatment mainly affected the surface of the samples and, consequently, was expected to lower the rupture force of dried seeds [34]. In CP-US-assisted hot-air drying, the contribution of CP pretreatment at temperatures of 35, 45, and 55 °C was no more than 11.94, 9.91, and 7.86%, respectively. In this drying variant, lower contributing effects were achieved, compared to ultrasound-assisted drying. The ultrasound power affected the internal tissues and created more porosities and intercellular space inside the seeds. Therefore, the destruction after ultrasound application became easier [62].

A combination of cold plasma pretreatment and hybrid ultrasound/convective drying is another possibility for drying caraway seeds. In this variant, the seeds with different CP pretreatment times were dried in the ultrasound-assisted system. For the seeds with a 25 s CP pretreatment time, the rupture force was evaluated in a range of 18.94–31.89 N, while for a CP exposure time of 50 s, the minimum and maximum rupture force were found as 30.59 N and 35.85 N, respectively. So, it is reasonable to conclude that the low exposure time and high ultrasound power provided more reducing effects, and the samples became crispier. By applying the pretreatment time of 25 s and the sonication power of 180 W, the results for rupture force were drastically affected and reduced by 45.92% at 35 °C. Decreases of 40.86 and 34.92% were also noted at the convective temperatures of 45 and 55 °C, respectively. Therefore, this drying program was found to be the best scheme for reducing the rupture force.

### 3.7. Total Phenolic Content (TPC)

**Figure 9:**
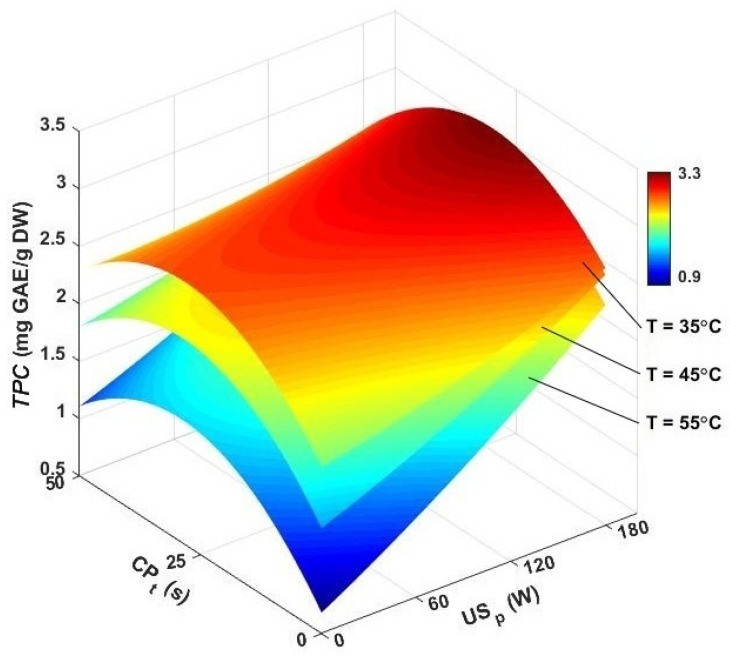
3D contour plots for TPC versus air temperature, cold plasma pretreatment, and ultrasonic power. *TPC*: total phenolic content, CP_t_: cold plasma exposure time, and US_p_: ultrasound power.

Figure 9 illustrates the influence of various research variables on the *TPC* of dried caraway seeds. In the single hot-air-drying domain, air temperature significantly affected the *TPC* of the dried seeds. In this program, the *TPC* of dried samples at 35 °C was 2.19 mg GAE/g, whereas at a higher temperature of 55 °C, the TPC decreased to 1.22 mg GAE/g. This clearly shows that higher drying temperatures significantly reduced *TPC* values in caraway seeds. Phenolic compounds are sensitive to heat. High temperatures can break down these compounds, leading to a reduction in *TPC* [65]. Previous studies have also confirmed that lower air temperatures can enhance the TPC of food materials [65,66,67].

Compared to pure hot-air drying, ultrasound assistance improved the *TPC*, with values ranging from 1.11 to 2.83 mg GAE/g. In ultrasound-assisted hot-air drying, the highest *TPC* (2.83 mg GAE/g) was achieved at a drying temperature of 45 °C with an ultrasound power of 180 W, while the lowest *TPC* (1.11 mg GAE/g) was recorded at 55 °C and 60 W. These results indicate that the effectiveness of ultrasound in preserving *TPC* was significantly affected by the drying temperature. At an ultrasound power of 180 W, *TPC* increased by 34.32, 25.32, and 17.28% at 35, 45, and 55 °C, respectively, compared to air drying alone. This suggests that the impact of sonication on *TPC* diminished at higher temperatures, as the reduced air density at these temperatures resulted in less ultrasound energy being transferred to the sample.

As can be seen in Figure 9, obtained values for *TPC* ranged from 1.47 to 3.86 mg GAE/g in ultrasound-assisted air drying of caraway seeds followed by CP pretreatment. The maximum value (3.86 mg GAE/g) was achieved with a 25 s pretreatment time, a drying temperature of 45 °C, and an ultrasound power of 180 W. The minimum *TPC* (1.47 mg GAE/g) was recorded with a 50 s CP time, followed by 55 °C and 60 W, as the drying conditions. It is noted from the results that a 50 s CP exposure time resulted in less improvement in TPC.

Earlier research indicated that plasma treatment likely leads to cell membrane degradation through inherent reactive species generated by the plasma. Interestingly, this process facilitates the extraction of phenolic compounds into the intercellular space [68]. The obtained results are in line with studies performed by Sarangapani et al. (2016), who found more phenolic compounds in plasma-treated parboiled rice flour [69]. More polyphenols were also noted in tomato-based beverages subjected to cold plasma processing [3]. On the contrary, Garofulić et al. (2015) reported degradation of polyphenol compounds, but they were affected by longer exposure to plasma treatment [70]. Other studies showed no effect of plasma on the anthocyanin content, which was linked by the authors to the voltage used for the plasma generator [71]. Short plasma treatment leads to the breakdown of chemical bonds, the dissociation of agglomerates or particles, and consequently, significant increases in anthocyanin and phenolic content. Thus, it can be stated that the process parameters of cold plasma treatment are fundamental in extracting polyphenols from food [70].

### 3.8. Total Flavonoid Content (TFC)

**Figure 10:**
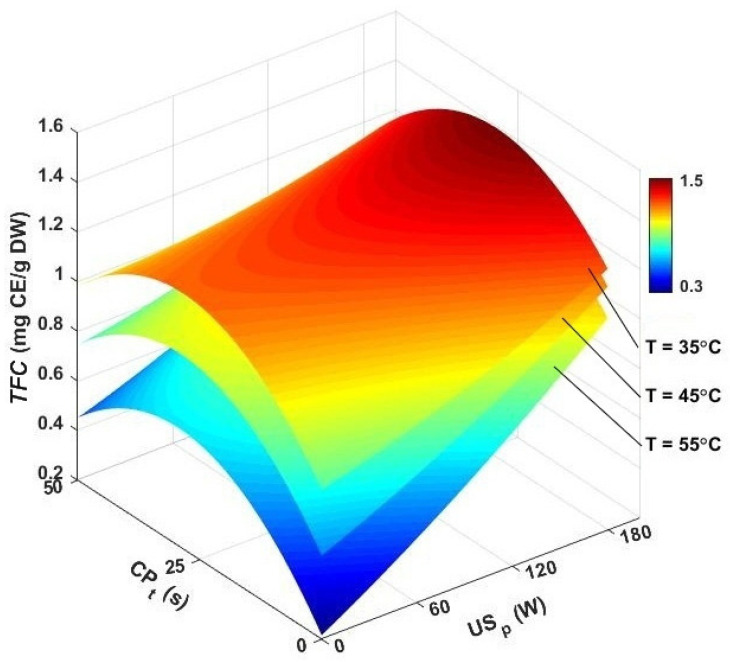
3D contour plots for TFC versus air temperature, cold plasma pretreatment, and ultrasonic power. *TFC*: total flavonoid content, CP_t_: cold plasma exposure time, and US_p_: ultrasound power.

Figure 10 shows the effect of different pretreatment and drying conditions on the *TFC* of dried caraway seeds. In pure hot-air drying, increasing the temperature caused the *TFC* to drop from 0.95 mg CE/g at 35 °C to 0.45 mg CE/g at 55 °C. Other studies also found that lower drying temperatures helped in preserving the *TFC* in dried food products. In hybrid ultrasound-assisted convective drying, *TFC* values ranged from 0.56 to 1.24 mg CE/g, with the highest TFC at 35 °C and 180 W, and the lowest at 55 °C and 60 W. Pretreating samples with CPt of 25 s before ultrasound-assisted drying increased TFC by 20.56, 54.48, and 11.40% at 35, 45, and 55 °C, respectively, compared to hot-air drying alone. Key results showed that *TFC* preservation by ultrasound assistance was higher than the CP pretreatment before hot-air drying. Combined application of CP and ultrasound had a synergistic effect on *TFC* preservation, with *TFC* values ranging from 0.54 to 1.79 mg CE/g. The best drying conditions were 25 s of CP pretreatment, 45 °C air temperature, and 180 W ultrasound power, while the least effective was 50 s of exposure, followed by drying at 35 °C with 60 W.

### 3.9. Antioxidant Activity

The *IC_50_* values (half-maximal inhibitory concentration) represent the concentration needed to inhibit 50% of the radicals. Therefore, a lower *IC_50_* value implies a more potent antioxidant in the extract [72]. In the pure air-drying domain, the *IC_50_* values were in the range of 32.33–43.26 μg/mL. The lowest value (32.33 μg/mL) belonged to the air temperature of 35 °C, while the highest was obtained at 55 °C. Higher air temperatures during hot-air drying can lead to a reduction in the antioxidant properties of food materials, which is reflected in higher *IC_50_* values. This means that higher temperatures can decrease the effectiveness of the antioxidants present in the food. The degradation of antioxidants at higher temperatures is due to the thermal breakdown of phenolic compounds and other bioactive substances. This breakdown reduces the overall antioxidant capacity of the dried product, making it less effective in inhibiting oxidative processes [72].

After ultrasound assistance during hot-air drying of caraway seeds, the *IC_50_* values ranged between 27.43 and 41.06 μg/mL. The lowest value (27.43 μg/mL) belonged to the temperature of 45 °C and ultrasound power of 180 W, while the highest one (41.06 μg/mL) was obtained at 55 °C and 60 W. Table 2 reveals that in the ultrasound-assisted air-drying domain, the interaction effect of air temperature and ultrasound power was significant on *IC_50_* values (*p*-value < 0.01); thus, by decreasing the air temperature and increasing the ultrasound power, the *IC_50_* values increased. The cavitation effect caused by ultrasound increased mass transfer rates and enhanced the extraction of bioactive compounds, which contributed to lower *IC_50_* values. This means that the food material retained more of its antioxidant capacity, making it more effective in inhibiting oxidative processes [3].

**Figure 11:**
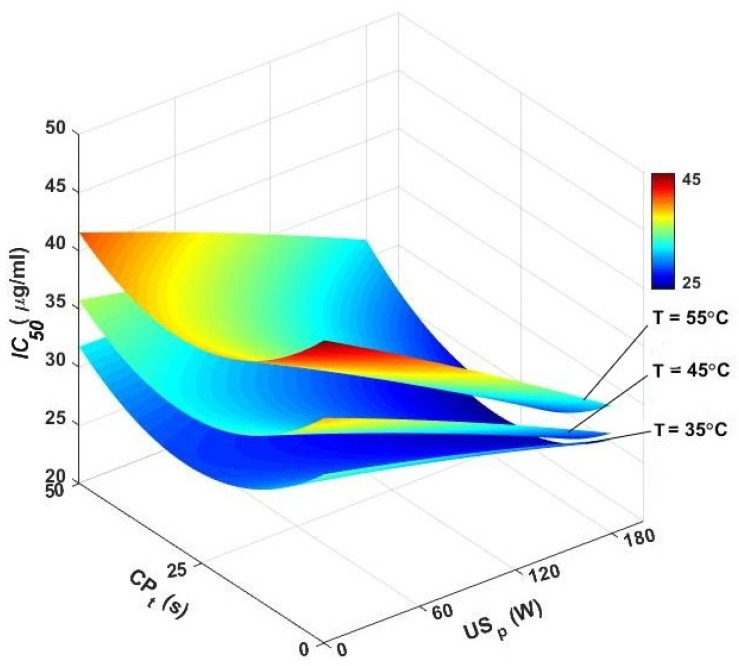
3D contour plots of DPPH scavenging activity (*IC_50_*) versus air temperature, cold plasma pretreatment, and ultrasonic power. *IC_50_*: DPPH scavenging activity, CP_t_: cold plasma exposure time, and US_p_: ultrasound power.

The current study demonstrated that the combined use of CP and ultrasound significantly increased the DPPH scavenging ability of the caraway seed extracts (Figure 11). CP pretreatment of caraway seeds for 25 s before ultrasound-assisted air drying at 45 °C and 180 W resulted in the lowest *IC_50_* value (19.66 μg/mL). This was 28.32% lower than that of the *IC_50_* value obtained in the same drying condition but without CP pretreatment (27.43 μg/mL). In each CP exposure time, by increasing the ultrasound power and decreasing the air temperature, the *IC_50_* value decreased, indicating more antioxidant activity. The mean *IC_50_* values of dried caraway seeds when pretreated with CPt of 25 s before ultrasound-assisted air drying were 24.55, 25.95, and 29.84% at 35, 45, and 55 °C, respectively, which were about 24, 34, and 32% lower than the values at the same drying temperatures but without CP and ultrasound application.

The effect of CP on the antioxidant activity of different extracts has been studied in several studies. Bao et al. (2020) examined the phenolic extracts obtained from tomato pomace and found that the CP enhanced the antioxidant activity by 30% [73]. In a similar study, Poomanee et al. (2021) applied the CP pretreatment to Luem Pua (LP) rice and reduced the measured *IC_50_* from 0.187 ± 0.025 mg/mL (for untreated samples) to 0.080 ± 0.002 mg/mL [65]. Hou et al. (2019) tested different treatment times and found that the antioxidant capacity of blueberry juice was crucially decreased by increasing the CP exposure time from 2 to 6 min [66]. Abedelmaksoud et al. (2022) showed the importance of CP pretreatment in preserving the antioxidant properties of fresh mango pulps [67]. Pogorzelska-Nowicka et al. (2021) examined 12 different herbs and showed that in 9 cases, the antioxidant capacity was remarkably improved by including cold plasma pretreatment on species. However, in three cases, namely, *Sanguisorba officinalis*, *Andrographis paniculata,* and *Polygonum aviculare,* exposure to CP showed no significant alterations for the extracts [74].

### 3.10. RSM-Based Modeling and Optimization

Via development of mathematical prediction models, it would be possible to determine the optimum drying conditions with minimum specific energy consumption and least adverse effects on the quality of dried samples. However, prior to any optimization process, it is necessary to investigate the accuracy of RSM models in the prediction of experimental measurements.

**Figure 12:**
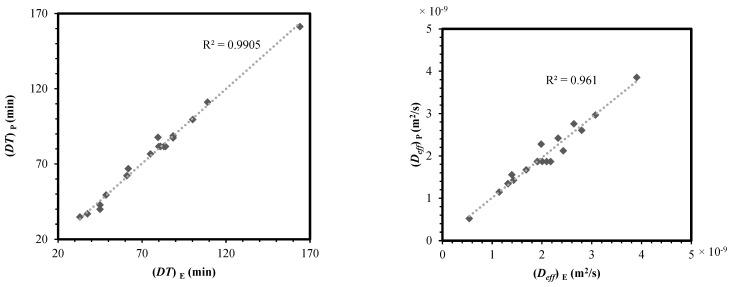

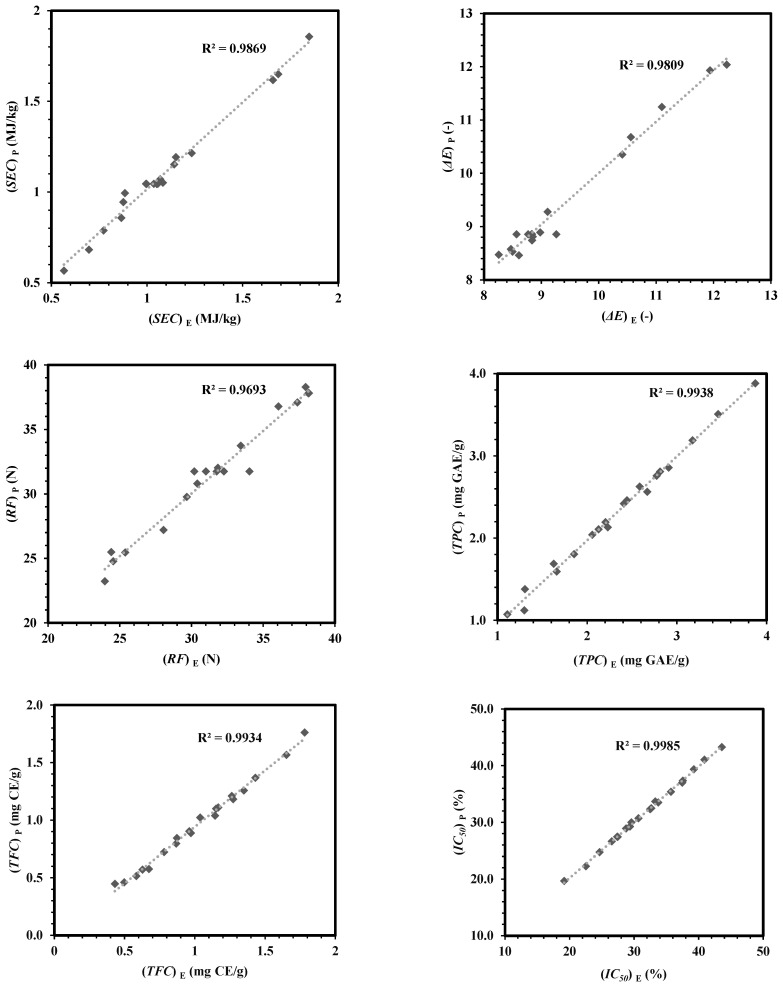
Relationship between experimental and RSM-based predicted data for the drying time, effective moisture diffusivity, specific energy consumption, total color change, and rupture force of caraway seeds. *DT*: drying time, *D_eff_*: diffusivity, *SEC*: specific energy consumption, *ΔE*: color change, *RF*: rupture force, *TPC*: total phenolic content, *TFC*: total flavonoid content, *IC_50_*: DPPH scavenging activity, P: predicted, and E: experimental.

Figure 12 compares the predictions of RSM-based models and those obtained in the experiments. As can be seen, the results of the numerical method and the outputs of tests for each response variable were in sufficient agreement. This clearly demonstrated the validity and accuracy of the polynomials proposed in Table 3. The error indices defined in Equations (9)–(12) were also evaluated, and their values are presented in Table 4. As an example, a range of 0.9677–0.9983 was found for *R*^2^. This confirmed the acceptability of RSM estimations performed for the assessment of the hybrid drying system.

To find the most efficient and appropriate drying conditions, the input variables were continuously varied, and the response variables were obtained by the polynomials derived by the RSM procedure. It should be noted that different criteria were used for the optimization of the drying process. For some output variables, such as the drying period, specific energy consumption, total color change, *IC_50_*, and the rupture force, it was desired to obtain a minimum value, while the effective moisture diffusivity, *TPC*, and *TFC* aimed to obtain the maximum. Three optimal drying conditions with desirability of 0.78 are presented in Table 5. These are the most efficient and economical processes for producing high-quality dried products.

## 4. Conclusions

In this study, the performance of a convective system for drying of caraway seeds was improved by cold plasma pretreatment and ultrasonic wave interference during the main drying process. The experimental results showed that by using cold plasma and ultrasound waves in the convective drying system, a major improvement was observed in the maximum state of the drying time. Consequently, the amount of consumed energy decreased, and fewer adverse effects were inflicted on the quality of the dried seeds. The combined application of CP and ultrasound was also found to be very beneficial for improving the levels of *TPC*, *TFC*, and antioxidant capacity of prepared extracts from the dried products. As the next step, the RSM approach was used for finding the optimal drying conditions. The analysis showed that for achieving the maximum desirability, the air temperature, the level of ultrasonic power, and the time of CP pretreatment should be selected as 35 °C, 180 W, and 20 s, respectively. An inventive approach that combines cold plasma pretreatment with ultrasound-assisted convective drying could be suggested as a substitute for single convective drying of seeds.

## Figures and Tables

**Table 1:** Experimental design and the results corresponding to each data point.

Independent Variables			Dependent Variables							
CP_t_ (s)	T (°C)	US_p_ (W)	*DT* (min)	*D_eff_* (m^2^/s)	*SEC* (MJ/kg)	*∆E* (-)	*RF* (N)	*TPC* (mg GAE/g)	*TFC* (mg CE/g)	*IC_50_* (%)
0	35	0	292	6.55 × 10^−10^	582.47	10.79	38.02	2.19	0.95	32.33
0	35	60	273	7.03 × 10^−10^	551.58	10.07	34.01	2.04	0.84	33.69
0	35	120	245	7.85 × 10^−10^	502.29	9.22	28.35	2.45	1.10	29.97
0	35	180	207	8.4 × 10^−10^	446.99	8.47	25.38	2.59	1.15	29.26
0	45	0	254	7.58 × 10^−10^	535.17	11.32	38.15	1.36	0.50	39.36
0	45	60	242	7.95 × 10^−10^	500.36	11.15	34.24	1.61	0.62	37.33
0	45	120	225	8.6 × 10^−10^	458.97	10.39	29.29	2.10	0.87	33.50
0	45	180	181	9.2 × 10^−10^	432.66	9.49	27.31	2.83	1.24	27.43
0	55	0	215	9.02 × 10^−10^	457.79	11.42	38.16	1.22	0.45	43.26
0	55	60	218	8.87 × 10^−10^	461.49	11.11	35.37	1.11	0.56	41.06
0	55	120	196	9.94 × 10^−10^	401.10	10.83	30.38	1.68	0.65	37.01
0	55	180	192	1.02 × 10^−9^	407.59	10.55	29.17	2.19	0.93	32.49
25	35	0	264	7.26 × 10^−10^	544.39	8.44	33.21	2.63	1.15	28.91
25	35	60	241	8.01 × 10^−10^	493.35	8.09	31.29	2.90	1.32	26.65
25	35	120	192	9.01 × 10^−10^	422.00	7.17	27.62	3.15	1.42	24.72
25	35	180	156	9.75 × 10^−10^	361.85	6.40	20.21	3.50	1.63	22.29
25	45	0	229	8.45 × 10^−10^	482.61	9.07	34.33	1.85	0.77	35.37
25	45	60	208	9.35 × 10^−10^	428.30	8.64	31.70	2.42	1.04	30.73
25	45	120	184	1.06 × 10^−9^	384.48	7.76	28.58	2.80	1.24	27.46
25	45	180	135	1.13 × 10^−9^	323.85	7.25	22.32	3.86	1.79	19.66
25	55	0	197	9.9 × 10^−10^	409.03	9.50	35.12	1.38	0.50	39.33
25	55	60	180	1.09 × 10^−9^	383.37	9.33	32.51	1.86	0.76	35.34
25	55	120	169	1.16 × 10^−9^	357.89	9.02	29.37	2.56	1.15	29.23
25	55	180	141	1.21 × 10^−9^	303.47	8.35	24.72	3.15	1.43	24.97
50	35	0	272	7.04 × 10^−10^	564.57	12.05	39.10	2.41	1.02	31.10
50	35	60	248	7.75 × 10^−10^	517.43	11.66	36.02	2.27	1.00	31.93
50	35	120	225	8.59 × 10^−10^	457.61	11.23	33.45	2.52	1.10	30.13
50	35	180	220	8.81 × 10^−10^	403.89	10.93	31.63	2.21	0.93	32.57
50	45	0	233	8.31 × 10^−10^	488.78	12.18	38.40	1.97	0.81	34.22
50	45	60	222	8.73 × 10^−10^	464.40	12.02	36.07	2.14	0.90	32.81
50	45	120	205	9.46 × 10^−10^	449.57	11.73	34.04	2.36	0.98	31.46
50	45	180	205	9.48 × 10^−10^	452.57	11.49	32.86	2.31	1.01	31.50
50	55	0	203	9.59 × 10^−10^	410.16	12.33	38.02	0.99	0.52	42.44
50	55	60	209	9.28 × 10^−10^	413.09	12.10	35.71	1.47	0.54	38.40
50	55	120	200	9.75 × 10^−10^	422.77	11.89	33.34	2.09	0.89	33.15
50	55	180	209	9.27 × 10^−10^	409.88	11.80	33.53	2.15	0.87	33.43

**Table 2:** ANOVA of second-order polynomials proposed for the response variables.

		Sum of Squares					
Input Variable	*df*	*DT* (min)	*D_eff_* (m^2^/s)	*SEC* (MJ/kg)	*TPC* (mg GAE/g)	*TFC* (mg CE/g)	*IC_50_* (%)
Model	9	1.225 × 10^5^ **	1.613 × 10^−18^ **	44,263.66 **	4.37 **	9.39 **	2779.43 **
*X*_1_	1	960.68 **	3.012 × 10^−20^ **	43,415.50 **	0.2983 *	0.0632 *	22.94 **
*X*_2_	1	32,004.50 **	7.379 × 10^−19^ **	6130.04 **	10.14 **	2.40 **	732.96 **
*X*_3_	1	50,189.70 **	4.452 × 10^−19^ **	1.525 × 10^5^	15.16 **	3.65 **	1037.27 **
*X*_1_ *X*_2_	1	481.33 ^ns^	1.145 × 10^−20^ *	684.67 ^ns^	0.0243 ^ns^	0.0097 ^ns^	8.88 ^ns^
*X*_1_ *X*_3_	1	2918.40 **	1.072 × 10^−20^ *	2603.65 *	0.6357 **	0.2183 **	61.91 **
*X*_2_ *X*_3_	1	8256.04 **	2.341 × 10^−20^ **	28,740.04 **	2.23 **	0.3769 **	183.75 **
*X*_1_^2^	1	26,555.67 **	3.520 × 10^−19^ **	65,793.34 **	10.47 **	2.63 **	709.81 **
*X*_2_^2^	1	573.63 *	1.016 × 10^−21 ns^	574.36 ^ns^	0.2586 *	0.0183 ^ns^	20.12 **
*X*_3_^2^	1	533.33 *	1.162 × 10^−21 ns^	227.93 ^ns^	0.1265 ^ns^	0.0339 ^ns^	1.78 ^ns^
R-Squared		0.9061	0.8786	0.9083	0.8912	0.8598	0.9174
Adj. R-Squared		0.8975	0.8675	0.8998	0.8812	0.8469	0.9098
C.V.%		5.33	5.28	4.83	9.93	12.99	4.94

** 0.001 ≤ *p*-value < 0.01 (very significant correlation), * 0.01 ≤ *p*-value ≤ 0.05 (significant correlation), ^ns^ insignificant. *DT*: drying time, *D_eff_*: diffusivity, *SEC*: specific energy consumption, *ΔE*: color change, *RF*: rupture force, *TPC*: total phenolic content, *TFC*: total flavonoid content, *IC_50_*: DPPH scavenging activity, *X*_1_ = CP_t_, *X*_2_ = T, and *X*_3_ = US_p_.

**Table 3:** Final second-order polynomials derived for each response variable in coded values.

Response Variable	Second-Order Polynomial Equations with Significant Coefficients
*DT* (min)	*DT* = 533.04 − 3.72*X*_1_ − 8.262*X*_2_ − 1.02*X*_3_ + 0.01*X*_1_*X*_2_ +0.02*X*_2_*X*_3_ + 0.05*X*_1_^2^ + 0.05*X*_2_^2^
*D_eff_* (m^2^/s)	*D_eff_* = (−2.40 + 1.39*X*_1_ + 1.99*X*_2_ + 0.25*X*_3_ − 0.02*X*_1_^2^) e^−11^
*SEC* (MJ/kg)	*SEC* = 771.69 − 6.27*X*_1_ − 2.91*X*_2_ − 1.95*X*_3_ + 0.02*X*_1_*X*_2_ + 0.03*X*_2_*X*_3_ + 0.09*X*_1_^2^ − 0.05*X*_2_^2^
*∆E* (-)	∆*E* = 7.18 − 0.17*X*_1_ + 0.13*X*_2_ − 0.02*X*_3_
*RF* (N)	*RF* = 35.80 − 0.28*X*_1_ + 0.08*X*_2_ − 0.10*X*_3_
*TPC* (mg GAE/g)	*TPC* = 2.17 + 0.06*X*_1_ + 0.03*X*_2_ − 0.01*X*_3_ − 0.001*X*_1_^2^ − 0.001*X*_2_^2^
*TFC* (mg CE/g)	*TFC* = 1.30 + 0.03*X*_1_ − 0.005*X*_2_ − 0.002*X*_3_
*IC_50_* (%)	*IC_50_* = 32.0 − 0.43*X*_1_ − 0.25*X*_2_ + 0.05*X*_3_ − 0.002*X*_1_*X*_2_ + 0.001*X*_1_*X*_3_ + 0.01*X*_1_^2^ + 0.01*X*_2_^2^

*DT*: drying time, *D_eff_*: diffusivity, *SEC*: specific energy consumption, *ΔE*: color change, *RF*: rupture force, *TPC*: total phenolic content, *TFC*: total flavonoid content, *IC_50_*: DPPH scavenging activity, *X*_1_ = CP_t_, *X*_2_ = T, and *X*_3_ = US_p_.

**Table 4:** The performance of the RSM model in predicting the drying parameters of caraway seeds.

Response Variable	Statistical Indicators			
	*RMSE*	*MAPE*	*MAE*	*R* ^2^
*DT* (min)	2.8148	3.1566	2.0756	0.9974
*D_eff_* (m^2^/s)	1.5 × 10^−10^	5.3189	1.11 × 10^−10^	0.9790
*SEC* (MJ/kg)	0.1291	2.8359	0.1365	0.9876
*∆E* (-)	0.1658	1.4387	0.1316	0.9932
*RF* (N)	0.7920	1.9026	0.5842	0.9947
*TPC* (mg GAE/g)	0.0629	2.5613	0.0465	0.9923
*TFC* (mg CE/g)	0.0659	6.4305	0.0604	0.9677
*IC_50_* (%)	0.2546	0.7075	0.2140	0.9983

*DT*: drying time, *D_eff_*: diffusivity, *SEC*: specific energy consumption, *ΔE*: color change, *RF*: rupture force, *TPC*: total phenolic content, *TFC*: total flavonoid content, and *IC_50_*: DPPH scavenging activity.

**Table 5:** Optimum drying conditions of caraway seeds, desirability, and the predicted responses at each optimum drying point.

No.	T (°C)	CP_t_ (s)	US_p_ (W)	*DT* (min)	*D_eff_* (m^2^/s)	*SEC* (MJ/kg)	*ΔE* (-)	*RF* (N)	*TPC* (mg GAE/g)	*TFC* (mg CE/g)	*IC_50_* (%)	Desirability
1	39.08	23.07	180	162.61	9.67 × 10^−10^	365.24	6.98	23.34	3.32	1.51	23.49	0.78
2	39.17	23.04	180	162.5	9.66 × 10^−10^	365.18	6.99	23.35	3.32	1.51	23.49	0.78
3	39.01	23.05	180	162.7	9.65 × 10^−10^	365.32	6.97	23.33	3.32	1.51	23.49	0.78

CP_t_: cold plasma exposure time, US_p_: ultrasound power, *DT*: drying time, *D_eff_*: effective moisture diffusivity, *SEC*: specific energy consumption, *ΔE*: color change, *RF*: rupture force, *TPC*: total phenolic content, *TFC*: total flavonoid content, and *IC_50_*: DPPH scavenging activity.

## Data Availability

The original contributions presented in the study are included in the article, further inquiries can be directed to the corresponding author.
